# Sex-Biased Sound Symbolism in English-Language First Names

**DOI:** 10.1371/journal.pone.0064825

**Published:** 2013-06-05

**Authors:** Benjamin J. Pitcher, Alex Mesoudi, Alan G. McElligott

**Affiliations:** 1 Biological and Experimental Psychology, School of Biological and Chemical Sciences, Queen Mary University of London, London, United Kingdom; 2 Department of Anthropology, Durham University, Durham, United Kingdom; University of Tasmania, Australia

## Abstract

Sexual selection has resulted in sex-based size dimorphism in many mammals, including humans. In Western societies, average to taller stature men and comparatively shorter, slimmer women have higher reproductive success and are typically considered more attractive. This size dimorphism also extends to vocalisations in many species, again including humans, with larger individuals exhibiting lower formant frequencies than smaller individuals. Further, across many languages there are associations between phonemes and the expression of size (e.g. large /a, o/, small /i, e/), consistent with the frequency-size relationship in vocalisations. We suggest that naming preferences are a product of this frequency-size relationship, driving male names to sound larger and female names smaller, through sound symbolism. In a 10-year dataset of the most popular British, Australian and American names we show that male names are significantly more likely to contain larger sounding phonemes (e.g. “Thomas”), while female names are significantly more likely to contain smaller phonemes (e.g. “Emily”). The desire of parents to have comparatively larger, more masculine sons, and smaller, more feminine daughters, and the increased social success that accompanies more sex-stereotyped names, is likely to be driving English-language first names to exploit sound symbolism of size in line with sexual body size dimorphism.

## Introduction

Ian Fleming noted that the name of his fictional hero, James Bond, was “*unromantic … yet very masculine*” [1]. The majority of English first names are specifically masculine or feminine, but why names are attributed to a specific sex is not clear. Previous studies have examined proximate factors governing name-gender assignment [2], but few have addressed its ultimate causes. The perceived masculinity or femininity of names may be due to sexually selected sound symbolism of size. Sexual selection has driven male-biased size dimorphism in over 45% of mammal species, including humans [3]. In general, taller men are perceived as more dominant and tend to be considered more attractive [4], [5]. Further, average to taller stature men have higher reproductive success than shorter men [6], [7]. Conversely, shorter and slimmer women are generally perceived as more attractive [8]–[10] and are more fecund [11], [12].

In many mammals, including humans, body size differences are revealed in the formant frequencies of an individual’s vocalisations. Formants are spectral peaks formed by the vocal tract selectively damping or enhancing resonant frequencies of the fundamental frequency of the voice. They are principally determined by the morphology of the vocal tract, which is constrained by surrounding skeletal structures and closely linked to body size [13]–[16]. Accordingly, the formant frequencies of mammal calls [14], [17], [18] and human speech [19] advertise body size, with lower and more closely spaced frequencies indicating larger body size. In human speech, vowel production depends on changes in tongue and lip position, and consequently the size and shape of the vocal tract, to alter the frequencies and dispersion of formants and thus vowel identity [13]. High front vowels (e.g. /i, e/, such as the /i/ in pit) typically have higher formants and greater dispersion, while low back vowels (e.g. /a, o/, such as the /<$>\raster(80%)="rg3"<$>/ in pot) have lower formants and dispersion [20], [21].

Sound symbolism is when a sound unit, such as a phoneme, goes beyond its linguistic function as a non-meaning-bearing unit to directly express a meaning [22], resulting in a systematic relationship between sound and meaning [23]. It may express a number of salient characteristics of an object or activity, including movement, size, shape, colour, and texture [23]. For example, the Japanese mimetics or ideophones *goro* and *koro* respectively mean “a heavy object rolling” and “a light object rolling” [24], [25]. The initial consonant indicates the size of the object, while the /r/indicates movement or rotation [24]. Sound symbolism has also been demonstrated in the classic kiki/bouba (also takete/baluma) experiments, in which the majority of participants associate the former word with a sharply inflected visual shape and the latter word with a more rounded shape [26]–[29].

**Figure 1:**
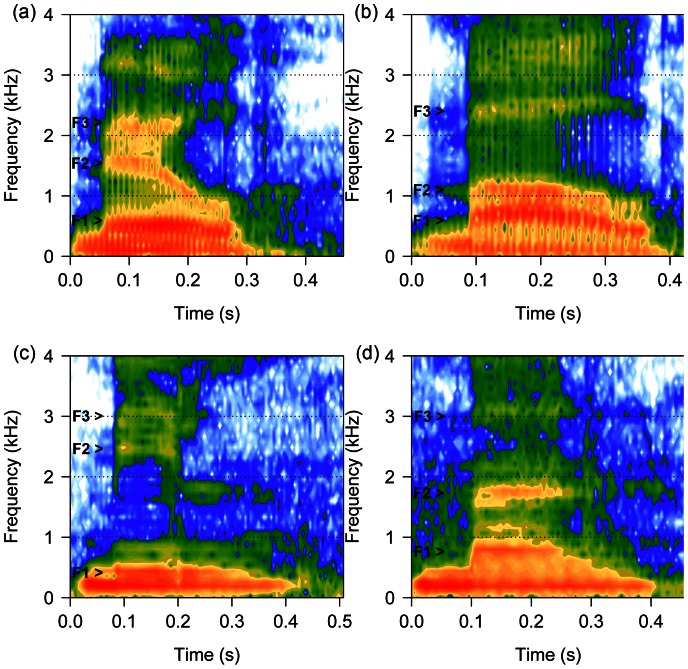
The relative position of formants in high front and low back vowels. Spectrograms showing a 175 cm tall male (a and b), and a 165 cm tall female (c and d) saying “mil” (high front vowel phoneme, spectrograms (a) and (c)) and “mal” (low back vowel phoneme, spectrograms (b) and (d)). The positions of the first three formants are labelled F1– first formant, F2– second formant, F3– third formant. Lower frequencies of F2 and lower dispersion between F1 and F2 can be seen in “mal” compared to “mil”. Overall, lower formant frequencies can be seen in the taller male voice compared to the shorter female voice. Spectrograms generated using Seewave [57], formants measured using Praat [58].

Examinations of sound symbolism of size have shown that people readily associate certain phonemes with different sizes [30], [31] and may be explained by a combination of articulatory, acoustic and biological factors [22]. Morton’s [32] “Motivational-Structural Rule Theory” proposed that animals should use harsh, low frequency sounds in hostile contexts and relatively high frequency, tonal sounds in friendly or appeasing contexts. This is because lower frequency vocalisations typically originate from larger vocal apparatuses and therefore larger, more threatening individuals. A continuation of this theory is the “Frequency Code” [30], [33], [34] in which listeners (particularly humans) associate higher frequencies with smaller vocalisers who are subordinate, submissive or non-threatening, and lower frequencies with larger vocalisers who are more dominant, threatening or aggressive. Ohala [30], [34] suggests that the frequency code interacts with the vocal resonances used to produce particular phonemes to result in sound symbolism of size in human speech. In humans, both adults [30], [35] and children as young as 4 months old [36] associate novel, nonsense words containing high front vowels (e.g. “mil”) with small sized objects, and words containing low back vowels (e.g. “mal”) with large-sized objects (Figure 1; Table 1). In vowel production, the frequency of the first formant (F1) is inversely related to the vowel height, while the frequency of the second formant (F2) is related to the front to back position of the tongue (backness); more forward is typically higher frequency [20], [21]. Therefore, similar to other mammalian vocalisations, the phonetic generalisation can be made that the expression of size in sound symbolism utilises vowel phonemes whose formant frequencies, and to some extent formant dispersion, vary inversely with the size being described [30]. Sound symbolism has been shown across several European, West African, Asian, South American and Native North American languages [22], [30], [34], [37], [38]. However, while the link has been demonstrated between phonemes and size, the implications of sound symbolism in first name choice have remained unexplored.

**Table 1:** Examples of the magnitude conveyed by vowel phonemes, from Johnson [31].

Phoneme	Example	Most often associated size
/I/	b*i*d	small
/i<$>\raster(70%)="rg2"<$>/	r*e*gal	small
/e/	s*e*ll	small
/a<$>\raster="rg6"<$>/	f*i*nd	small
/<$>\raster(75%)="rg3"<$>/	sm*o*ck	small
/<$>\raster(75%)="rg5"<$>/	m*u*st	small
/u<$>\raster(70%)="rg2"<$>/	st*u*pid	large
/<$>\raster(70%)="rg1"<$>/	b*i*rd	large
/æ/	br*a*nd	large
/e<$>\raster(75%)="rg6"<$>/	fr*a*il	large
/a<$>\raster(70%)="rg4"<$>/	c*o*w	large
/a<$>\raster(70%)="rg2"<$>/	m*o*ttled	large
/o/	b*o*ne	large

Here we propose that naming preferences have developed as a product of the sound symbolic frequency code and preferred sexual traits. Sexual selection has acted on the body, vocal system and perceptual abilities of humans resulting in sexual size dimorphism and perceptible size related cues in vocalisations [3], [19], [39], [40]. English-language naming preferences may then follow body size preferences leading to a name-sex dimorphism paralleling the observed body size dimorphism, with names containing vowels with lower formant frequencies and dispersion favoured for males, and vowels with higher formant frequencies and dispersion favoured for females. We analysed the 50 most popular first names for males and females retrieved from the public databases of (i) England and Wales, (ii) New South Wales (Australia) and (iii) the United States for the 10 years between 2001 and 2010, inclusive. We hypothesize that male names should be more likely to contain a large sounding stressed syllable, while female names should contain a smaller sounding stressed syllable. This would result in male names being perceived as more masculine and female names as more feminine, thus increasing their perceived attractiveness.

## Materials and Methods

Name frequency statistics for male and female children born between 2001 and 2010 were obtained from government databases (Table 2). The top 50 first names for each sex in each year were collated. Rankings were kept as they were published and therefore alternate spellings of names and similar sounding names were included as separate entries and not grouped (e.g. “Madison” and “Maddison”). The proportion of all children given one of the top 50 first names during the 10 years was determined by comparing the number of children given a name in the top 50 and the total number of births recorded each year.

**Table 2:** Popular name data sources.

Source	Region	URL^1^	Data usage statement
Social Security Online	United States of America (Excl. territories)	http://www.ssa.gov/OACT/babynames/	Public domain.
NSW Registry of Births Deaths and Marriages	New South Wales, Australia	http://www.bdm.nsw.gov.au/births/popularBabyNames.htm	Data for the most popular names for Boys and Girls for the period of 2001 to 2010 is reproduced with the permission of the NSW Registry of Births Deaths & Marriages for and on behalf of the Crown in and for the State of New South Wales. It is subject to Crown copyright.
Office for National Statistics	England and Wales, United Kingdom	http://www.ons.gov.uk	Source: Office for National Statistics licenced under the Open Government Licence v1.0.www.nationalarchives.gov.uk/doc/open-government-licence/open-government-licence.htm

1 Specific information about the data collection methods used by each source can be found on their websites. The rankings were kept as they were published and therefore alternate spellings of names and similar sounding names were included as separate entries and not grouped (e.g., “Madison” and “Maddison”). Data accessed 2012 Jan 31.

Names were transcribed into the International Phonetic Alphabet to represent their constituent phonemes. To avoid bias, transcriptions were taken from Jones [41]. Alternate American English pronunciations were used for US names if available. If a pronunciation was not provided (<8% of names), pronunciation information was obtained from online sources, such as: www.babynames.co.uk or www.wikipedia.org.

The assignment of a vowel phoneme as either sound symbolically large or small was determined from previous studies [31], [35]. Using the findings of Johnson [31] the phonemes were divided as follows, small:/i, I, i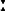, e, aI, 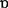, 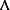/, and large: /u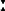, 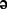, 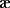, eI, a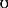, a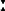, o/. Any other phonemes were classified as either high front or low back and assigned the corresponding size.

In total, 3000 entries, comprising 112 unique male and 151 female names, were examined. For each name, the vowel phoneme of the primary stressed syllable was determined. The frequency of vowel phonemes was calculated within each year for each sex. The mean number of large and small phonemes was determined by dividing the sum of the frequencies of each size category by the number of phonemes in each category. Comparisons between these means were made using SPSS 16.0 for Windows. Within each sex, a chi-squared test with a null hypothesis of equal distribution was used to examine the distribution of large and small phonemes. Between the sexes, t-tests were used to compare the mean number of names for large and small phonemes. Data are presented as mean ± S.E.

## Results

**Figure 2:**
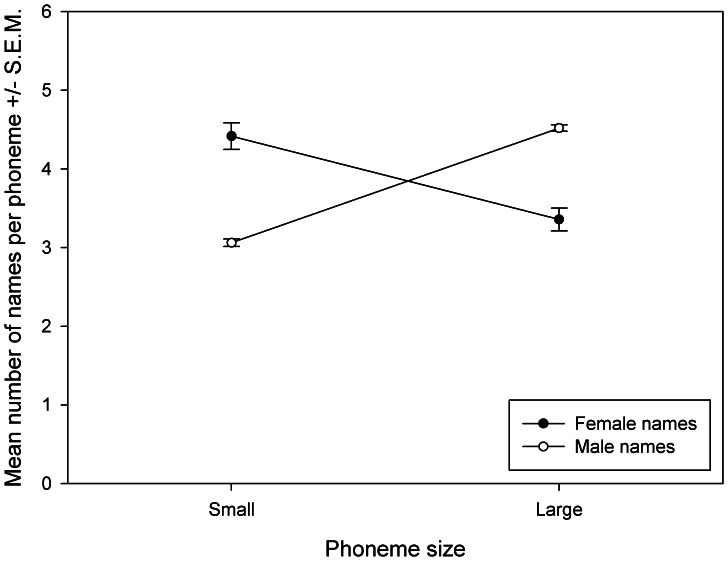
The mean number of names per phoneme for each phoneme size category. **Closed circles = Female names, Open circles = Male names.**

Collectively, the top 50 names from each region were given to almost 15 million babies, or 30.6% of all recorded births, during the 10 years. When considering the vowel phoneme of the stressed syllable of each name, we found that male names were significantly more likely to have a large sounding vowel phoneme than a small sounding phoneme (Small: 3.08±0.027 names/phoneme, Large: 4.53±0.019 names/phoneme; χ^2^ (1, N = 229) = 8.84, p = 0.003; Figure 2). Conversely, female names were significantly more likely to have a small stressed syllable vowel phoneme than a large phoneme (Small: 4.61±0.081 names/phoneme, Large: 3.54±0.072 names/phoneme; χ^2^ (1, N = 238) = 6.72, p = 0.01; Figure 2). As a group, the male names were much more likely to contain large stressed syllable vowel phonemes than the female names (*t*_234_ = 14.94, p<0.001), while female names contained significantly more small stressed syllable vowel phonemes than male names (*t*_222_ = 15.17, p<0.001). Therefore male names are more likely to be sound symbolic of large sizes (e.g. “Thomas”) while female names are more likely to be symbolic of smaller sizes (e.g. “Emily”).

## Discussion

We investigated the relationship between sound symbolism of size and the phonemic content of popular English-language first names. Instead of an arbitrary association between names and sexes, preference for gender-size congruity in the sound of names appears to have driven a size symbolic relationship between names and sexes, with male names generally sounding larger than female names. We found that male names are far more likely to contain a vowel phoneme in the stressed syllable that is sound symbolic of larger size, while female names are more likely to be symbolic of smaller size. English-language first names therefore follow the sexual size dimorphism observed in human body size [3], [42].

In males, names that feature lower formant frequencies and dispersion are likely to be perceived as larger and therefore favoured. This is likely to influence both the perceived competitive ability and attractiveness of an individual, as formant position is negatively correlated with upper body strength [43], and formant frequencies and formant dispersion are negatively correlated with body size [19], [44], [45]. Studies of human speech have shown that male voices with lower formant frequencies and dispersion are perceived as more masculine, as well as physically and socially dominant [39]. In females, the bias towards higher formant frequencies and smaller sounding names potentially advertises the femininity of the individual, because greater formant dispersion is correlated with shorter vocal tracts and smaller body size [19], [46]. Typically, in Western societies, women who are shorter, slimmer and have smaller waists relative to their hips are perceived as more attractive by men and have higher reproductive success [8]–[12]. Men prefer more feminine voices, particularly in short-term relationships, and are highly attentive to levels of formant dispersion [47]. Women also pay attention to formant dispersion in other women’s voices and perceive more feminine voices as more attractive and flirtatious [47]. This suggests that indicators of femininity in women’s voices are likely to be under both inter- and intrasexual selection. Thus, a male name that increases the perceived size of its bearer is likely to be favoured over one that indicates a more diminutive stature, while in females names that are perceived as smaller are likely to be regarded as more feminine.

While it is usually true that parents choose the name of their child rather than a child naming itself, parents will stand to gain indirect fitness benefits by increasing their offspring’s attractiveness and success. Parents may not actively seek a large or small sounding name for their child, but instead are likely to show an unconscious preference for either a more masculine or feminine name to suit their child’s sex. Parental expectations for a child are likely to be reflected in name choices. The perceived connotations of names are widespread. For instance, children reliably associate the holders of particular male names with either more active or passive behaviours, such as “Baxter” with running and “Aldwin” with colouring pictures [48]. Further, when students were asked to predict the success of a person at an occupation, more masculine or feminine names were associated with an expectation of higher success at correspondingly masculine or feminine occupations, while mismatched names and occupations had a much lower expectation of success [49]. These expected behavioural patterns associated with names might both govern the rewards conferred for complying with expectations, and the names chosen by parents for their children.

It has previously been shown that baby name choices are subject to cultural drift, as if they were being chosen at random akin to neutral genetic alleles [50], [51]. Here we have shown that there is a non-random selective force causing male and female names to diverge, although there may be cultural drift within male and female name pools. Bentley et al. [50] suggest that first names may not be totally unconstrained and that observed differences in mutation rate between sexes might be the product of constraint; preference for gender appropriate sound symbolism may account for some of that constraint.

Similar patterns of sexual size dimorphism of names may potentially be found in other languages. In Huambisa, a speech community of the Jivaroan language family in north central Peru, smaller bird and fish species, less than 25 cm in length, are more likely to have names containing high, front /i/ phonemes, whereas the names of larger species are more likely to contain /a, u/ [37]. This association between species size and vowel phoneme content has also been found in the unrelated languages Wayamp? (Tupian), Apalái (Cariban), and Tzeltal (Myan) [37]. While our result indicates that size dimorphism in English-language first names fits the observed attractiveness tendencies of Western societies, we would not expect the observed relationship to be true in societies where smaller and slimmer women are not perceived as more attractive. In South African Zulu societies, for example, women with higher body mass indices and larger waists relative to hips are considered more attractive, because body fat is potentially a symbol of prosperity [52]. We therefore predict that female names symbolic of larger sizes might be favoured in such societies. Similarly, the observed relationship may not occur where other cultural conventions or preferences exist, such as the use of prefixes or suffixes to define gender in some languages [53].

The observed size dimorphism in names, and particularly larger male names, shows similarities to the sexually selected breeding vocalisations of other mammals, where males advertise body size when calling. Because of the fundamental similarities in the vocal production apparatus [16], [19], similar correlations between formant frequencies and dispersion with age, body size, dominance and/or reproductive success are found across mammal groups (e.g. cervids [17], [18], [54], pinnipeds [55], primates [14]). In many size dimorphic species, the vocal structures have been modified through selection to advertise the body size of the caller. This has been proposed as the origin of the descended larynx in some cervids and other mammals, including humans [56]. It is likely that as a result of the inter- and intrasexual selection, which has shaped the human voice [40], preference for gender-size congruity in the sounds of names chosen by parents for their children has resulted in larger sounding names for males and smaller sounding names for females.

To conclude, this study has provided the first indication that in both sexes, preferences for gender-size congruity in the sounds of names has resulted in a population of names that display sound symbolism consistent with the attractive traits of their respective sexes. Further examinations of sound symbolism of size in names across other languages and using experimental presentations are necessary to determine the prevalence of this hypothesised preference. Finally, we suggest that this preference for gender-size appropriate sounding names is likely to be an ultimate cause for the sex attribution of first names.

## References

[1] Caplen RA (2010) Shaken & stirred: The feminism of James Bond. Bloomington, IN, USA: Xlibris, Corp.

[2] Cassidy KW, Kelly MH, Sharoni LJ (1999) Inferring gender from name phonology. Journal of Experimental Psychology 128: 362–381.

[3] Lindenfors P, Gittleman JL, Jones KE (2007) Sexual size dimorphism in mammals. In: Fairbairn DJ, Blanckenhorn WU, Székely T, editors. Sex, Size and Gender Roles: Evolutionary Studies of Sexual Size Dimorphism. Oxford, UK: Oxford University Press.

[4] Buunk AP, Park JH, Zurriaga R, Klavina L, Massar K (2008) Height predicts jealousy differently for men and women. Evolution and Human Behavior 29: 113–139.

[5] Pawlowski B, Jasieńska G (2005) Women’s preferences for sexual dimorphism in height depend on menstrual cycle phase and expected duration of relationship. Biological Psychology 70: 38–43. 16038772 10.1016/j.biopsycho.2005.02.002

[6] Pawlowski B, Dunbar RIM, Lipowicz A (2000) Tall men have more reproductive success. Nature 403: 156. 10.1038/35003107 10646589

[7] Stulp G, Pollet TV, Verhulst S, Buunk AP (2012) A curvilinear effect of height on reproductive success in human males. Behavioral Ecology and Sociobiology 66: 375–384. 22389549 10.1007/s00265-011-1283-2 PMC3277695

[8] Brown WM, Price ME, kang J, Pound N, Zhao Y, et al (2008) Fluctuating asymmetry and preferences for sex-typical bodily characteristics. Proceedings of the National Academy of Sciences, USA 105: 12938–12943. 10.1073/pnas.0710420105 PMC2529114 18711125

[9] Singh D (1993) Adaptive significance of female physical attractiveness: Role of waist-to-hip ratio. Journal of Personality and Social Psychology 65: 293–307. 8366421 10.1037//0022-3514.65.2.293

[10] Tovée MJ, Reinhardt S, Emery JL, Cornelissen PL (1998) Optimum body-mass index and maximum sexual attractiveness. The Lancet 352: 548. 10.1016/s0140-6736(05)79257-6 9716069

[11] Jasieńska G, Ziomkiewicz A, Ellison PT, Lipson SF, Thune I (2004) Large breasts and narrow waists indicate high reproductive potential in women. Proceedings of the Royal Society B 271: 1213–1217. 15306344 10.1098/rspb.2004.2712 PMC1691716

[12] Stulp G, Verhulst S, Pollet TV, Buunk AP (2012) The effect of female height on reproductive success is negative in western populations, but more variable in non-western populations. American Journal of Human Biology 24: 486–494. 22410858 10.1002/ajhb.22252

[13] Fant G (1960) Acoustic theory of speach production The Hague, Netherlands: Mouton.

[14] Fitch WT (1997) Vocal tract length and formant frequency dispersion correlate with body size in rhesus macaques. Journal of the Acoustical Society of America 102: 1213–1222. 9265764 10.1121/1.421048

[15] Taylor AM, Reby D (2010) The contribution of source-filter theory to mammal vocal communication research. Journal of Zoology 280: 221–236.

[16] Titze IR (1994) Principles of vocal production. Englewood Cliffs, NJ, USA: Prentice-Hall.

[17] Reby D, McComb K (2003) Anatomical constraints generate honesty: acoustic cues to age and weight in the roars of red deer stags. Animal Behaviour 65: 519–530.

[18] Vannoni E, McElligott AG (2008) Low frequency groans indicate larger and more dominant fallow deer (*Dama dama*) males. PLoS One 3: e3113. 18769619 10.1371/journal.pone.0003113 PMC2518835

[19] Fitch WT, Giedd J (1999) Morphology and development of the human vocal tract: a study using magnetic resonance imaging. Journal of the Acoustical Society of America 106: 1511–1522. 10489707 10.1121/1.427148

[20] Hillenbrand J, Getty LA, Clark MJ, Wheeler K (1995) Acoustic characteristics of American English vowels. Journal of the Acoustical Society of America 24: 175–184. 10.1121/1.411872 7759650

[21] Peterson GE, Barney HL (1952) Control methods used in a study of the vowels. Journal of the Acoustical Society of America 24: 175–184.

[22] Nuckolls JB (1999) The case for sound symbolism. Annual Review of Anthropology 28: 225–252.

[23] Hinton L, Nichols J, Ohala JJ (1994) Sound-symbolic processes. In: Ohala JJ, Hinton L, Nichols J, editors. Sound Symbolism. Cambridge, UK: Cambridge University Press.

[24] Kita S (2008) World-view of protolanguage speakers as inferred from semantics of sound symbolic words: A case of Japanese mimetics. In: Masataka N, editor. The Origins of Language, Unraveling Evolutionary Forces. Tokyo, Japan: Springer.

[25] Ahlner F, Zlatev J (2010) Cross-modal iconicity: a cognitive semiotic approach to sound symbolism. Sign Systems Studies 38: 298–348.

[26] Köhler W (1929) Gestalt Psychology (1st Edition). New York, NY, USA: Liveright.

[27] Köhler W (1947) Gestalt Psychology (2nd Edition). New York, NY, USA: Liveright.

[28] Davis R (1961) The fitness of names to drawings: a cross-cultural study in Tanganyika. British Journal of Psychology 52: 259–268. 13720232 10.1111/j.2044-8295.1961.tb00788.x

[29] Ramachandran VS, Hubbard EM (2001) Synaesthesia - A window into perception, thought and language. Journal of Consciousness Studies 8: 3–34.

[30] Ohala JJ (1994) The frequency code underlies the sound-symbolic use of voice pitch. In: Ohala JJ, Hinton L, Nichols J, editors. Sound Symbolism. Cambridge, UK: Cambridge University Press.

[31] Johnson RC (1967) Magnitude symbolism of English words. Journal of Verbal Learning and Verbal Behavior 6: 508–511.

[32] Morton EW (1977) On the occurrence and significance of motivation-structural rules in some bird and mammal sounds. American Naturalist 111: 855–869.

[33] Ohala JJ (1980) The acoustic origin of the smile. Journal of the Acoustical Society of America 68: S33.

[34] Ohala JJ (1984) An ethological perspective on common cross - language utilization of F0 of voice. Phonetica 41: 1–16. 6204347 10.1159/000261706

[35] Sapir E (1929) A study in phonetic symbolism. Journal of Experimental Psychology 12: 225–239.

[36] Peña M, Mehler J, Nespor M (2011) The role of audiovisual processing in early conceptual development. Psychological Science 22: 1419–1421. 21960249 10.1177/0956797611421791

[37] Berlin B (1994) Evidence for pervasive synesthetic sound symbolism in ethnozoological nomenclature. In: Ohala JJ, Hinton L, Nichols J, editors. Sound Symbolism. Cambridge, UK: Cambridge University Press.

[38] Chuenwattanapranithi S (2008) Encoding emotions in speech with the size code – a perceptual investigation. Phonetica 65: 210–230. 19221452 10.1159/000192793

[39] Puts DA, Hodges CR, Cárdenas RA, Gaulin SJC (2007) Men’s voices as dominance signals: vocal fundamental and formant frequencies influence dominance attributions among men. Evolution and Human Behavior 28: 340–344.

[40] Puts DA, Jones BC, DeBruine LM (2012) Sexual selection on human faces and voices. Journal of Sex Research 49: 227–243. 22380590 10.1080/00224499.2012.658924

[41] Jones D (2006) English Pronouncing Dictionary. Cambridge, UK: Cambridge University Press.

[42] Manning JT (1995) Fluctuating asymmetry and body weight in men and women: implications for sexual selection. Ethology and Sociobiology 16: 145–153.

[43] Puts DA, Apicella CL, Cárdenas RA (2012) Masculine voices signal men’s threat potential in forager and industrial societies. Proceedings of the Royal Society B 279: 601–609. 21752821 10.1098/rspb.2011.0829 PMC3234546

[44] Bruckert L, Liénard J-S, Lacroix A, Kreutzer M, Leboucher G (2006) Women use voice parameters to assess men’s characteristics. Proceedings of the Royal Society B 273: 83–89. 16519239 10.1098/rspb.2005.3265 PMC1560007

[45] Evans S, Neave N, Wakelin D (2006) Relationships between vocal characteristics and body size and shape in human males: An evolutionary explanation for a deep male voice. Biological Psychology 72: 160–163. 16280195 10.1016/j.biopsycho.2005.09.003

[46] Collins SA, Missing C (2003) Vocal and visual attractiveness are related in women. Animal Behaviour 65: 997–1004.

[47] Puts DA, Barndt JL, Welling LLM, Dawood K, Burriss RP (2011) Intrasexual competition among women: Vocal femininity affects perceptions of attractiveness and flirtatiousness. Personality and Individual Differences 50: 111–115.

[48] Bruning JL, Husa FT (1972) Given names and stereotyping. Developmental Psychology 7: 91.

[49] Bruning JL, Polinko NK, Zerbst JI, Buckingham JT (2000) The effect on expected job success of the connotative meanings of names and nicknames. Journal of Social Psychology 140: 197–201. 10808642 10.1080/00224540009600459

[50] Bentley RA, Hahn MW, Shennan SJ (2004) Random drift and culture change. Proceedings of the Royal Society B 271: 1443–1450. 15306315 10.1098/rspb.2004.2746 PMC1691747

[51] Mesoudi A, Lycett SJ (2009) Random copying, frequency-dependent copying and culture change. Evolution and Human Behavior 30: 41–48.

[52] Tovée MJ, Swami V, Furnham A, Mangalparsad R (2006) Changing perceptions of attractiveness as observers are exposed to a different culture. Evolution and Human Behavior 27: 443–456.

[53] Alford RD (1988) Naming and identity: a cross-cultural study of personal naming practices. New Haven, CT, USA: HRAF Press.

[54] Briefer E, Vannoni E, McElligott AG (2010) Quality prevails over identity in the sexually selected vocalisations of an ageing mammal. BMC Biology 8: 35. 20380690 10.1186/1741-7007-8-35 PMC2858104

[55] Sanvito S, Galimberti F, Miller EH (2007) Vocal signalling of male southern elephant seals is honest but imprecise. Animal Behaviour 73: 287–299.

[56] Fitch WT, Reby D (2001) The descended larynx is not uniquely human. Proceedings of the Royal Society B 268: 1669–1675. 11506679 10.1098/rspb.2001.1704 PMC1088793

[57] Sueur J, Aubin T, Simonis C (2008) Seewave: a free modular tool for sound analysis and synthesis. Bioacoustics 18: 213–226.

[58] Boersma P, Weenink D (2012) Praat: doing phonetics by computer [Computer program]. Version 5.3.10. http://www.praat.org/. Accessed 2012 Mar 14.

